# A rare case report of prosthetic loosening secondary to diffuse non-pigmented villonodular synovitis after bilateral total knee arthroplasty

**DOI:** 10.3389/fsurg.2025.1709479

**Published:** 2025-12-02

**Authors:** Ke Cai, Wei Yan, Leqin Lin, Lei Li, Hong Yu, Haina Jiang, Hongjiang Jiang

**Affiliations:** 1 First Clinical Medical College, Shandong University of Traditional Chinese Medicine, Jinan, China; 2 Department of Bone and Joint Surgery, Wendeng Orthopaedic Hospital of Shandong Province, Weihai, Shandong, China; 3 Department of Pathology, Wendeng Orthopaedic Hospital of Shandong Province, Weihai, Shandong, China

**Keywords:** nonpigmented villonodular synovitis, prosthesis loosening, total knee arthroplasty, synovial hyperplasia, longitudinal outcomes, revision arthroplasty

## Abstract

**Background:**

Nonpigmented villonodular synovitis (non-PVNS) is a benign yet locally aggressive proliferative disorder affecting the synovium. The occurrence of non-PVNS following knee joint replacement is exceedingly rare.

**Case presentation:**

This study investigated synovial mechanisms driving aseptic loosening in bilateral total knee arthroplasty failure over a 14-year period. Radiographic and pathologic analysis tracked sequential failure progression bilaterally. Initial radiographs documented progressive left-knee loosening (2009–2016). Subsequent intraoperative and histologic evaluation (H&E staining) confirmed synovial hyperplasia without PVNS features as the primary mechanism. Post-revision imaging showed immediate left-knee stabilization. However, long-term follow-up revealed sustained left-knee stability (2019) but emergent right-knee loosening (2023), demonstrating interlimb heterogeneity and recurrence risk. Pathologic examination of the revised right knee identified an analogous synovial hyperplasia mechanism (non-PVNS).

**Conclusion:**

Final post-revision radiographs validated successful stabilization following both revisions. This longitudinal bilateral case uniquely demonstrates synovial hyperplasia as a replicable driver of aseptic loosening independent of PVNS, highlights heterogeneous progression kinetics and recurrence risk between limbs despite unilateral intervention, and confirms the consistent efficacy of revision arthroplasty for this specific pathology.

## 1 Background

Pigmented Villonodular Synovitis (PVNS) is a rare benign proliferative disorder characterized by locally aggressive involvement of synovial joints, tendon sheaths, and extra-articular bursae, with the knee being the predominant site of occurrence (1). Pathologically, it manifests as nodular synovial hyperplasia with extensive hemosiderin deposition and is classified into localized and diffuse subtypes. Malignant transformation and distant metastases are exceptionally rare events (2). Histopathological diagnosis remains the gold standard, demonstrating synovial cell hyperplasia in superficial and subsynovial layers under microscopic examination. At low magnification, distinct villonodular architecture is observed, while high magnification reveals diffuse stromal cell proliferation admixed with fibroblastic tissue, multinucleated giant cells, xanthoma-like cells, lymphocytes, and variable hemosiderin deposits. The non-PVNS exhibits identical pathological features except for the absence of hemosiderin deposition (3). Paradoxically, while studies indicate a lower incidence of PVNS compared to non-PVNS following knee arthroplasty, published literature documents substantially more PVNS cases than non-PVNS cases postoperatively. To date, only a single case of extensive non-PVNS after total knee arthroplasty has been reported in the English medical literature (4).

Total knee arthroplasty (TKA) is a highly successful intervention for end-stage osteoarthritis, reliably restoring function and alleviating pain with generally favorable long-term outcomes (5). Despite its efficacy, a subset of TKAs inevitably fail, requiring complex revision surgeries that impose significant burdens on both patients and healthcare resources. Aseptic loosening, the progressive loss of implant fixation without overt infection, represents a predominant cause of late TKA failure (6). While wear-particle-induced inflammation is the widely accepted primary mechanism (7), the precise biological events governing the initiation, progression, and potential recurrence of loosening remain incompletely characterized, hindering the development of effective preventative and therapeutic approaches.

The dominant paradigm attributes aseptic loosening to an inflammatory cascade triggered by phagocytosis of wear debris (e.g., polyethylene, metal) by macrophages within the peri-implant tissues (8). This process releases pro-inflammatory cytokines (TNF-α, IL-1β, IL-6), promoting osteoclast activation and bone resorption around the implant (9). The synovium-derived membrane forming at the bone-implant interface is central to this reaction (10). However, considerable variability exists in the progression rate and pattern of aseptic loosening, even between similar implants within the same patient. Key questions remain unanswered regarding the drivers of this heterogeneity, the potential contribution of specific synovial pathologies distinct from classic wear-particle reactions, and the long-term fate of the underlying pathology following revision surgery (11). Addressing these knowledge gaps is crucial for improving prognostication and optimizing patient management strategies.

Synovial pathologies, particularly hyperplasia, are increasingly implicated in inflammatory arthritides and prosthetic joint failure. While synovial proliferation is frequently observed histologically adjacent to loose implants, its role as a primary driver vs. a secondary phenomenon remains contested (12). Conditions such as PVNS starkly illustrate the destructive potential of hyperplastic synovium, leading to aggressive local bone destruction (13). However, synovial hyperplasia implicated in TKA failure often lacks the characteristic histological features of PVNS; the pathobiology and clinical significance of this non-PVNS synovial hyperplasia as a primary driver of loosening are inadequately understood.

Crucially, longitudinal data capturing the natural history and progression kinetics of such specific synovial pathologies within the TKA context are exceptionally scarce. This gap is particularly evident for bilateral cases, which present a unique opportunity to examine limb-specific progression and the behavior of the pathology post-intervention. Does the synovial process responsible for failure recur in the revised joint? Does it subsequently manifest in the contralateral limb? What are the long-term revision outcomes when arthroplasty specifically addresses isolated hyperplastic synovitis? Defining the specific mechanism (e.g., non-PVNS synovial hyperplasia) and its spatiotemporal dynamics (e.g., interlimb progression heterogeneity, latency to contralateral failure) is essential for refining diagnostic criteria, prognostic models, risk stratification (particularly in bilateral TKA), and ultimately, management approaches tailored to this distinct failure mechanism.

This study addresses these critical questions through a unique, comprehensively documented 14-year longitudinal analysis of sequential bilateral TKA failure. Integrating detailed serial radiographic tracking with definitive histopathological assessment, we demonstrate that localized synovial hyperplasia, explicitly distinct from PVNS, constituted the primary mechanism of aseptic loosening in both prostheses. Our findings uniquely reveal contrasting failure progression between limbs and a significant latency period to contralateral failure despite successful revision of the index side. Furthermore, we provide direct evidence confirming the sustained efficacy of revision arthroplasty targeting this specific synovial pathology. This case offers novel and substantial insights into non-PVNS synovial hyperplasia as a replicable driver of bilateral TKA failure, its heterogeneous kinetics, and the positive outcomes achievable with definitive surgical management.

## 2 Case presentation

**Figure 1:**
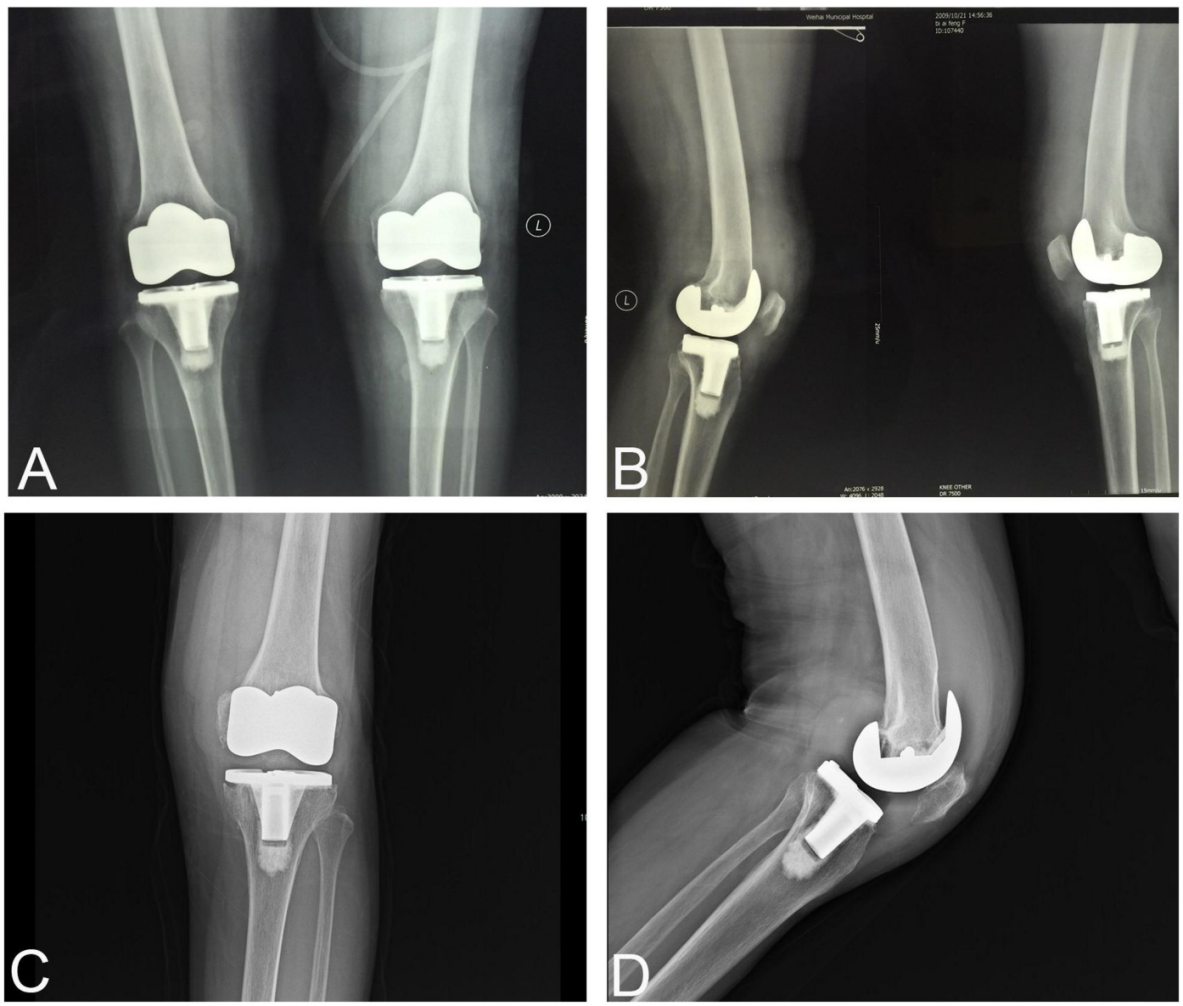
**(A)** Anteroposterior radiograph after the initial total knee arthroplasty in 2009. **(B)** Lateral radiograph after the initial surgery in 2009. **(C)** Anteroposterior radiograph after left knee symptom aggravation in 2016. **(D)** Lateral radiograph after left knee symptom aggravation in 2016.

A 75-year-old female underwent staged bilateral total knee arthroplasty (TKA) for primary osteoarthritis in 2009, with no preoperative or intraoperative evidence of pigmented villonodular synovitis (PVNS). Immediate postoperative radiographs confirmed optimal prosthesis positioning without loosening signs (Figures 1A,B), and the patient achieved uncomplicated recovery with full range of motion.

At 5 years postoperatively (2014), the left knee developed acute-onset swelling and pain without precipitating factors, accompanied by restricted mobility. Symptoms demonstrated partial responsiveness to conservative measures including rest and pharmacotherapy. One year later (2015), recurrent symptom exacerbation occurred, with arthrocentesis revealing yellow serous fluid exhibiting negative bacterial culture. Although revision surgery was medically indicated, the patient declined intervention. By postoperative year 7 (2016), progressive refractory symptoms necessitated tertiary care evaluation. Physical examination documented significant joint effusion (positive ballottement sign), localized warmth over the joint line, reproducible tenderness along medial/lateral compartments, and restricted arc of motion (0° extension, 10° flexion contracture, maximum 100° flexion). Radiographic and computed tomography (CT) imaging demonstrated femoral and tibial component loosening with periprosthetic osteolysis, consistent with aseptic failure (Figures 1C,D).

**Figure 2:**
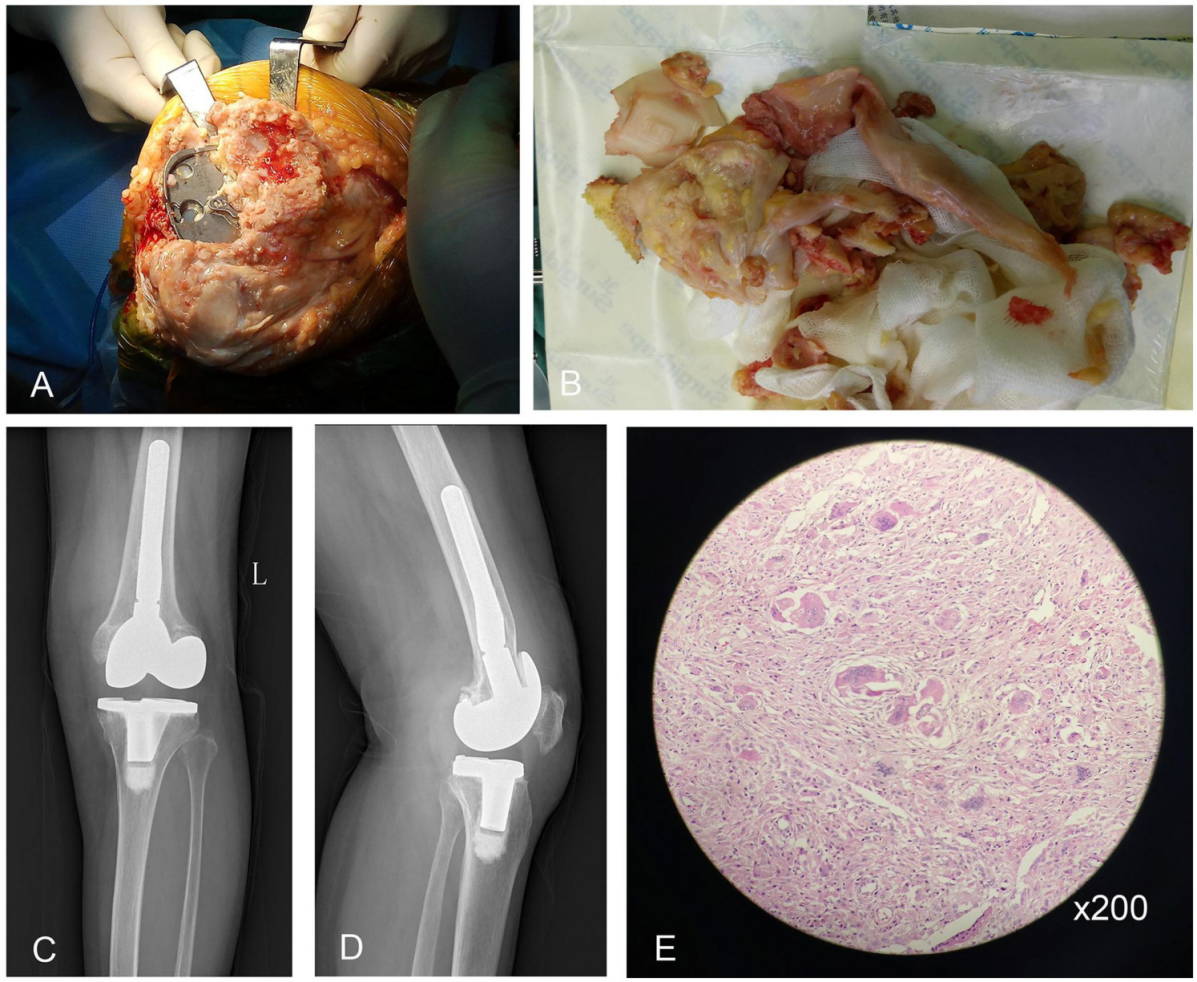
**(A)** Intraoperative view of loosened femoral prosthesis with proliferative synovium. **(B)** Resected papillary and nodular synovium. **(C)** The anteroposterior radiograph after left knee revision arthroplasty. **(D)** The lateral radiograph after left knee revision arthroplasty. **(E)** Histopathology confirming non-PVNS (synovial papillae with giant cells, no hemosiderin).

Left knee revision arthroplasty (2016) revealed unanticipated pathological findings: Diffuse synovial hyperplasia with papillomatous projections and nodular formations, accompanied by distinct tan-brown periprosthetic tissue discoloration (Figure 2A). Intraoperative assessment identified gross loosening of the femoral component with substantial metaphyseal bone loss, while the tibial component remained securely fixed. Given minimal polyethylene insert wear, isolated tibial polyethylene exchange was performed with retention of the original tibial tray. Macroscopic inspection of resected specimens demonstrated tissue surfaces covered with variably sized (short-to-long) papillomatous nodules (Figure 2B). The models of the prostheses implanted during this revision surgery were the ZIMMER NexGen® Complete Knee Solution, specifically:Legacy® Knee-Constrained Condylar (LCCK), Femoral Component Option, Size C Left; Stem Extension Offset, 13 mm Diameter × 100 mm Length (Combined Length 145 mm); Distal Femoral Augment Block Precoat, Size C 10 mm Augment With Screw; Posterior Femoral Augment Block Precoat, Size C 5 mm Augment With Screw; Legacy® Knee-Posterior Stabilized LPS-Flex Articular Surface, Size C D 14 mm Height. PALACOS® R bone cement (Heraeus Medical GmbH) was used for component fixation during the revision procedure. Reconstruction employing femoral/tibial extended stems and metallic augments achieved stable fixation. Immediate postoperative radiographs confirmed appropriate component positioning and mechanical alignment (Figures 2C,D). Histopathological analysis (Figure 2E) confirmed synovium-lined papillary fronds containing stromal infiltrates of multinucleated giant cells, histiocytes, and lymphocytes, with definitive absence of hemosiderin deposition—pathognomonic for non-PVNS inflammatory arthropathy.

**Figure 3:**
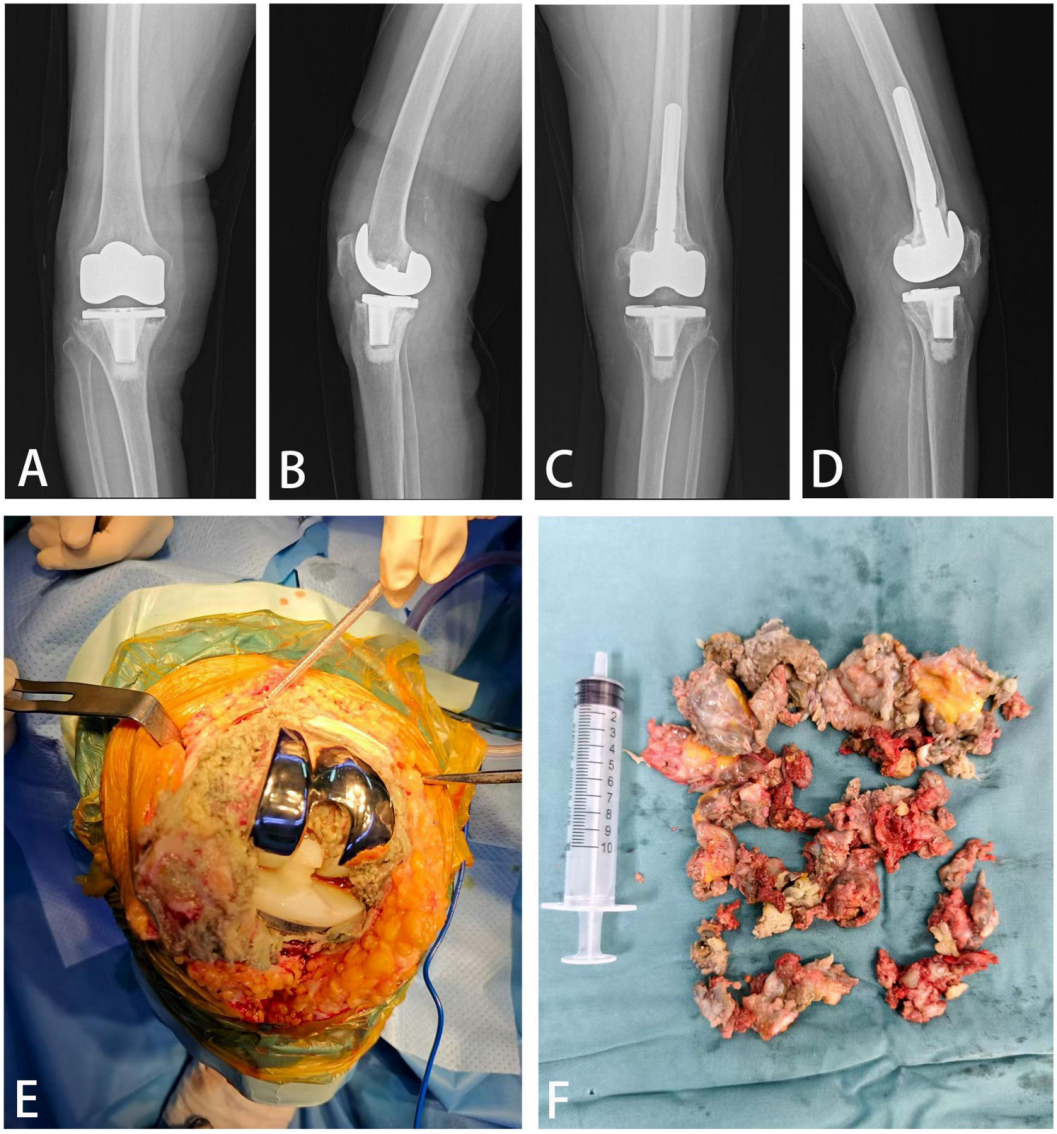
**(A)** The 2023 anteroposterior x-ray after initial total knee arthroplasty. **(B)** The 2023 lateral x-ray of the right knee. **(C)** The 2023 follow-up anteroposterior x-ray after left knee revision surgery. **(D)** The 2023 lateral follow-up x-ray of the left knee. **(E)** Intraoperative view of synovial hyperplasia. **(F)** Resected nodular synovium.

Strikingly symmetrical disease progression manifested in the contralateral knee at 13 years post-primary arthroplasty (2022). Initial symptom onset mirrored the left knee trajectory: acute unprovoked swelling/pain partially responsive to conservative management, followed by progressive worsening at 1-year post-onset (2023). Physical examination revealed identical findings of effusion (ballottement-positive), joint line tenderness, and equivalent motion restriction (0° −10° −100°). To exclude systemic rheumatic diseases, the patient underwent comprehensive physical examination (assessing major weight-bearing joints) and serological rheumatologic screening, all of which yielded normal results. Comparative radiography demonstrated new-onset tibial component loosening with subsidence, progressive osteolytic lesions in the distal tibia relative to 2016 baselines, and concomitant joint space narrowing (Figures 3A,B).

**Figure 4:**
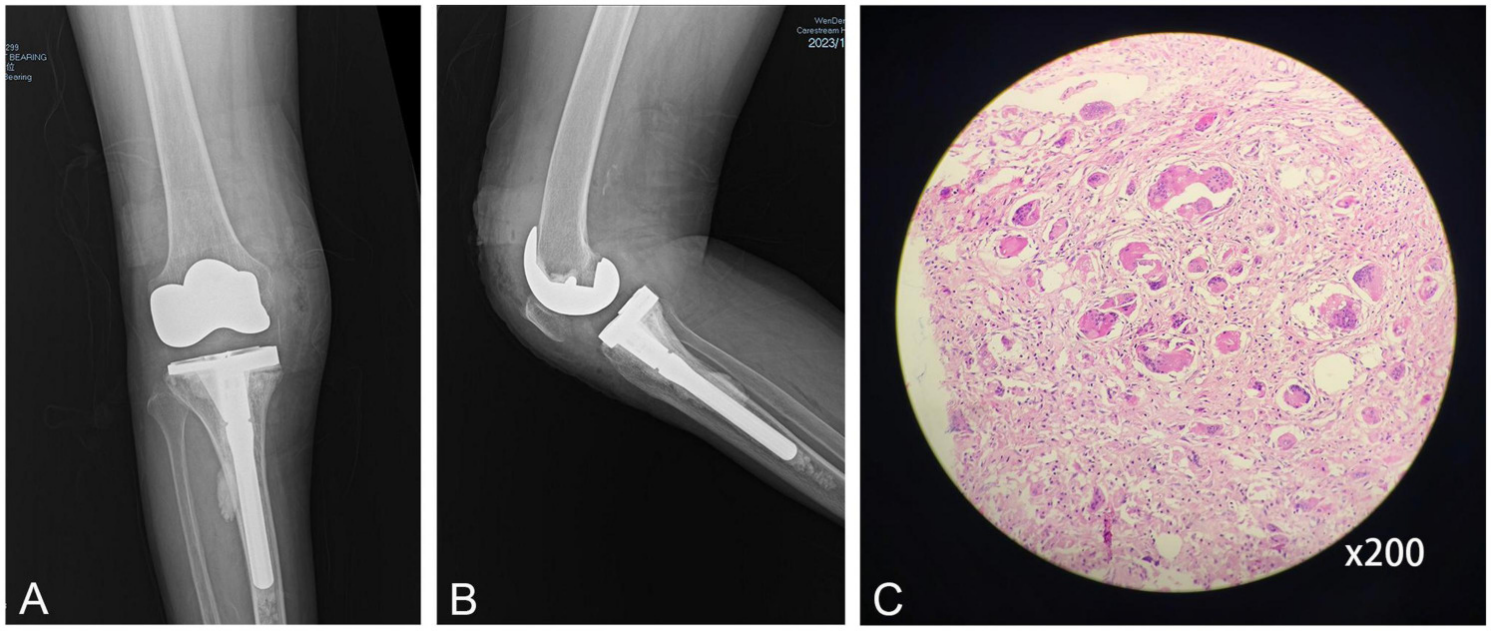
**(A)** The anteroposterior radiograph after right knee revision arthroplasty. **(B)** The lateral radiograph after right knee revision arthroplasty. **(C)** Histopathology replicating non-PVNS features.

Right knee revision surgery (2023) corroborated pathological symmetry: Copious tan-brown synovial fluid and exuberant brown nodular synovitis were evident (Figure 3E). While the femoral component remained well-fixed, the tibial component exhibited gross instability with substantial medial tibial bone loss. The excised hyperplastic synovial tissue was fixed in formalin, embedded in paraffin, and sectioned. After routine hematoxylin-eosin staining, it was evaluated by a senior orthopedic pathologist for histopathological assessment. Histopathological assessment of resected hyperplastic synovial tissue demonstrated identical microscopic architecture to the left knee specimen—featuring synovial papillae infiltrated by giant cells and lymphocytes without hemosiderin deposition, reconfirming the non-PVNS diagnosis (Figures 3F, 4C). The specific models of the prostheses used in this revision surgery were as follows: the ZIMMER NexGen® Complete Knee Solution series: Stemmed Tibial Component Precoat, Size 2 For Cemented Use Only; Stem Extension Straight, 12 mm Diameter × 100 mm Length (Combined Length 145 mm); Legacy® Knee-Posterior Stabilized (PS) Prolong® Highly Crosslinked Polyethylene LPS-Flex Articular Surface, Size C D 14 mm Height (Use With Plate 1, 2); Tibial Block Precoat, Size 2, 5 mm Thickness With 2 Screws. PALACOS® R bone cement (Heraeus Medical GmbH) was used for component fixation during the revision procedure. Final postoperative radiographs documented successful bilateral reconstruction with optimal component alignment and fixation (Figures 4A,B), and no signs of loosening or other common indications, achieving definitive therapeutic resolution.

## 3 Discussion

In this case report we observed that both knees of a single patient who underwent bilateral TKA ultimately failed due to diffuse non-PVNS. The left knee presented with progressive loosening six years after the index arthroplasty, whereas the right knee failed fourteen years postoperatively. In both episodes, revision with radical synovectomy and component replacement led to symptomatic relief and stable fixation. Histological assessment of synovial tissue showed villous hyperplasia with multinucleated giant cells but without hemosiderin deposition, features that distinguish diffuse non-PVNS from classic PVNS (14).

This sequential bilateral manifestation offers insights into the heterogeneity of synovial proliferative disorders after knee arthroplasty. PVNS and non-PVNS are rare complications after TKA and are mostly reported as single unilateral cases. Diffuse PVNS typically presents with recurrent haemarthrosis and early implant loosening due to pigmented synovial proliferation with hemosiderin deposits (15), whereas non-PVNS lacks pigment yet shows papillary hypertrophy with giant cells and macrophages (16). Our case expands this spectrum by demonstrating that diffuse synovial hyperplasia without hemosiderin can recur in contralateral joints many years apart. The left knee revision at seven years and the right knee revision at fourteen years highlight a markedly variable latency, suggesting that local biological factors rather than mechanical stress alone drive disease progression. The absence of significant wear debris at revision, together with the villous architecture and multinucleated giant cells, argues against mechanical metallosis and instead supports an intrinsic synovial proliferative process akin to non-PVNS (17). Some authors propose that diffuse synovial hyperplasia may arise from over-expression of growth factors such as epidermal growth factor (EGF) and transforming growth factor-α (TGF-α); interfacial membranes retrieved at revision arthroplasty exhibit markedly higher numbers of EGF and TGFα positive cells than primary synovium, and these cytokines stimulate osteoclast mediated bone resorption (18). Synovitis has also been identified as an independent predictor of aseptic loosening, whereas synovial cell hyperplasia predicts other modes of failure (19). Our case confirms that diffuse synovial hyperplasia can act as the primary driver of aseptic loosening even in the absence of overt metallosis or infection (20).

In classic PVNS, a chromosomal translocation between COL6A3 and CSF1 results in over expression of colony-stimulating factor-1 (CSF1), which recruits CSF1 receptor positive macrophages to the synovial lining (21). This neoplastic driver explains the pigmented appearance and often aggressive course, and targeted inhibition of the CSF1R pathway with pexidartinib or vimseltinib has produced objective responses in up to 40%–72% of patients (22, 23). In contrast, diffuse non-PVNS does not show CSF1 translocation and lacks hemosiderin; it is thought to be reactive, driven by chronic inflammation or microtrauma. The pathophysiology of aseptic loosening typically involves wear particles that activate macrophages and polarize them toward an M1 phenotype, leading to secretion of pro-inflammatory cytokines such as tumour necrosis factor-α, interleukin-1β and interleukin-6 and subsequent osteolysis (24, 25). Our histology did not reveal extensive polyethylene or metal debris, and the green pigment associated with cement wear seen in cement-induced synovitis was absent (26), suggesting a distinct aetiology from the wear particle–induced macrophage activation described in revision arthroplasty. Instead, the villous hyperplasia with giant cell clusters parallels findings from diffuse non-PVNS case reports (4, 16) and replicates across both knees. The sequential nature also distinguishes our patient from previous PVNS cases after TKA, which are mostly unilateral and often present earlier. Kia and colleagues reported PVNS presenting with recurrent hemarthrosis four years after TKA (27), whereas other case series have described multiple cases of PVNS causing loosening within the first decade after surgery (28). In our patient, there was no haemarthrosis and the second knee failed much later, emphasising that diffuse non-PVNS may have a more indolent yet progressive course.

Both knees recovered well after revision with radical synovectomy and component replacement. Such positive outcomes are consistent with reported experience in cases of synovial hypertrophy and PVNS after arthroplasty. Arthroscopic or open synovectomy and polyethylene exchange relieve pain and mechanical symptoms associated with patellofemoral clunk syndrome and synovial hypertrophy (29, 30). Pollock and colleagues described arthroscopic removal of intercondylar synovial tissue causing soft-tissue impingement, with all patients experiencing symptom resolution (31). In PVNS, cruciate-retaining TKA yields satisfactory mid-term outcomes, with implant survivorship of 90% at seven years and comparable functional scores to osteoarthritis controls (32). Even though postoperative stiffness is more frequent in PVNS patients, revision for loosening is rare (32). Our patient also demonstrated stable implants with no recurrence of synovial proliferation during the observation period. This suggests that timely surgical intervention can arrest the destructive process and restore function, even in diffuse non-PVNS.

Wear debris remains the predominant cause of aseptic loosening. Metallic and polyethylene particles induce a foreign body granulomatous response with macrophage and giant cell infiltration; periprosthetic tissues in aseptic loosening produce high levels of pro-inflammatory cytokines and chemokines (33). T-lymphocytes can constitute up to 10% of the interface and may modulate the foreign body reaction (34), and hypersensitivity reactions to metal debris occasionally trigger persistent effusion and need for revision (34). However, polymer particles alone elicit a non-specific macrophage response, and there is limited evidence for adaptive immunity against polymethylmethacrylate (35). In metallosis and metal-induced synovitis, the synovium is infiltrated by black granular pigment due to metallic particles and often associated with early patellar component failure (17). The absence of such pigment in our specimen supports a diagnosis of non-PVNS. Synovial hyperplasia may also result from metal hypersensitivity, but this phenomenon affects fewer than 1% of patients (34) and typically manifests as dermatitis and pain rather than villous hypertrophy. Consequently, our case underscores the existence of a distinct, non-pigmented synovial proliferative process that can compromise prosthesis stability independent of wear.

Methodologically, a key strength of our study lies in the integrated radiologic-histologic trajectory analysis spanning 14 years. This approach captured critical temporal nuances in the evolution of synovial hyperplasia and its biomechanical consequences on fixation—dynamics inherently missed by large-scale registry analyses like Lombardi et al., which, while invaluable for population-level trends, lack the granularity needed to unravel complex tissue-level mechanisms (36). This underscores the enduring value of meticulous, longitudinal clinical-pathological assessment for generating profound mechanistic insights into complex biological processes underlying implant failure.

The recognition of non-PVNS synovial hyperplasia as a primary etiological agent necessitates a significant reconceptualization within the field of arthroplasty failure (37). This demands corresponding advancements in clinical strategy and implant design philosophy. A critical implication concerns the use of adjuvant radiotherapy post-synovectomy (38). While effective for aggressive, recurrent PVNS or diffuse tenosynovial giant cell tumor, our findings suggest it should be approached with greater caution and selectivity in non-PVNS cases (39, 40). Its application should be reserved rigorously for scenarios exhibiting documented high recurrence risk factors (e.g., extensive or inaccessible disease burden, high proliferative index on histology) (41). This restraint is paramount because the risk profile shifts significantly; the primary concern in the arthroplasty context is not merely synovial recurrence but the potential for radiation-induced loosening (42, 43). Radiation can induce osteonecrosis and impair osseointegration, thereby contributing to implant instability—a failure mechanism distinct from, and potentially additive to, the original synovial pathology (44, 45). This necessitates a careful risk-benefit calculus, weighing the potential reduction in synovial recurrence against the increased risk of radiation-induced osteolysis and component destabilization.

Beyond radiotherapy, our findings underscore the critical importance of optimizing intraoperative debridement to achieve truly radical synovectomy (46). Techniques such as intraoperative frozen section analysis to verify the completeness of resection, particularly at marginal zones and the posterior capsule, could prove invaluable in minimizing the risk of recurrence originating from biologically active residual tissue. Routine synovial biopsy during revision arthroplasty—especially for unexplained loosening—could mitigate underdiagnosis, as non-PVNS lacks overt features like metallosis or infection.

As with any single case, our study has limitations. Because only one patient was analysed, confounding factors such as genetic predisposition, systemic inflammatory conditions, or subtle mechanical stresses could not be disentangled. We must honestly state that due to limitations in our hospital's laboratory facilities, specific PCR testing for atypical pathogens was not performed. Noting that although conventional microbiological tests were negative, the possibility of low-virulence or atypical infections detectable by PCR cannot be entirely ruled out. We did not perform genetic testing for CSF1 gene translocation or assess CSF1 or growth factor levels in the synovial tissue, limiting our ability to definitively categorise the lesion as purely reactive. Detailed family, genetic, and psychosocial histories were not obtained. Magnetic resonance imaging was not used to monitor the contralateral knee prior to failure, so early synovial changes may have been missed. We also did not evaluate serum biomarkers of inflammation or wear particle load, and we could not explore associations between systemic factors such as diabetes or immunological status and disease progression. Finally, follow-up after the second revision is still limited; longer surveillance is necessary to determine whether recurrence or further involvement will occur.

The rarity of diffuse synovial hyperplasia after TKA means that its natural history and optimal management remain poorly defined. Future work should establish multicentre registries to capture the incidence, time course and risk factors for non-PVNS after arthroplasty. Prospective imaging with ultrasound or MRI may detect early villous changes and guide timely intervention. Molecular characterisation of synovial tissue using genomic and transcriptomic approaches could clarify whether non-PVNS is a reactive process driven by growth factors such as EGF and TGF-α or a neoplastic lesion distinct from PVNS. Because CSF1/CSF1R signalling drives PVNS and targeted inhibitors such as vimseltinib achieve significant tumour regression and functional improvement (23), similar targeted approaches might be explored for diffuse synovial proliferative disorders. Moreover, immunomodulatory therapies that alter macrophage polarisation from pro-inflammatory a M1 phenotype toward reparative M2 phenotypes (12) and growth factor inhibitors could attenuate synovial hyperplasia and prevent osteolysis. Finally, implant design and surgical technique should aim to minimise wear debris and microtrauma, thereby reducing the inflammatory milieu that may trigger synovial proliferation. Education of patients and clinicians about late-onset synovial disease after TKA is essential to enable early recognition and management.

## 4 Conclusions

We definitively demonstrate that localized synovial hyperplasia, explicitly distinct from PVNS, acts as the primary driver of aseptic loosening in sequential bilateral knee arthroplasty failure. This causal role was conclusively established through histopathological confirmation (H&E staining) of synovial hyperplasia in both revised prostheses, corroborated by serial radiographic documentation of progressive loosening preceding each revision. Critically, the longitudinal trajectory revealed asymmetric progression: revision arthroplasty achieved durable stabilization (>7 years) in the index limb, whereas the initially unaffected contralateral joint manifested *de novo* loosening after a prolonged latency period (>6 years), unequivocally demonstrating the risk of asynchronous failure recurrence despite successful unilateral intervention.

These findings fundamentally alter the paradigm for arthroplasty failure by elevating non-PVNS synovial hyperplasia from a secondary phenomenon to a primary etiological driver. This necessitates revising diagnostic protocols to incorporate synovial biopsy during revision surgery for contralateral joint risk stratification. Beyond orthopedics, the extended latent period (>6 years) preceding contralateral failure highlights a localized joint-specific immune privilege enabling insidious pathology progression, informing the development of biomaterials targeting synovial dysregulation. Clinically, these results compel a transformation in surveillance strategies: patients with bilateral TKAs require dedicated, staggered imaging surveillance (e.g., biennial MRI) to detect subclinical contralateral synovitis, enabling early intervention. This paradigm shift aligns with value-based care by redirecting resources from costly revisions toward prevention.

Immediate validation necessitates establishing a multicenter registry by 2026 to quantify the prevalence of non-PVNS synovial hyperplasia using standardized immunohistochemical phenotyping (e.g., CD68+/CD3+immunophenotyping) while allowing regional governance, similar to existing arthroplasty registries, and correlating synovial fluid cytokine dynamics with failure latency. Critical next steps involve employing finite element analysis to model the biomechanical impact of hyperplastic synovium stress on implant interfaces, identifying potential recurrence triggers. A significant caveat identified in this case is the substantial (>6-year) latency to contralateral manifestation, indicating that standard radiographic surveillance likely fails to detect evolving subclinical synovitis. Consequently, an urgent priority is developing validated synovial molecular biomarkers and quantitative advanced imaging parameters to predict and preempt contralateral failure prior to overt radiographic loosening.

## Data Availability

The original contributions presented in the study are included in the article/Supplementary Material, further inquiries can be directed to the corresponding author.
